# Polymeric Dental Nanomaterials: Antimicrobial Action

**DOI:** 10.3390/polym14050864

**Published:** 2022-02-22

**Authors:** Pavel Yudaev, Vladimir Chuev, Bogdan Klyukin, Andrey Kuskov, Yaroslav Mezhuev, Evgeniy Chistyakov

**Affiliations:** 1 Mendeleev University of Chemical Technology of Russia, Miusskaya Sq., 9, 125047 Moscow, Russia; yudaevpavel5@gmail.com (P.Y.); bourne4432@gmail.com (B.K.); a_n_kuskov@mail.ru (A.K.); valsorja@mail.ru (Y.M.); 2 Belgorod National Research University, Pobedy Street, 85, 308015 Belgorod, Russia; mxpion@rambler.ru

**Keywords:** nanomaterials, dentistry, nanoparticles, antibacterial activity, antimicrobial action

## Abstract

This review aims to describe and critically analyze studies published over the past four years on the application of polymeric dental nanomaterials as antimicrobial materials in various fields of dentistry. Nanoparticles are promising antimicrobial additives to restoration materials. According to published data, composites based on silver nanoparticles, zinc(II), titanium(IV), magnesium(II), and copper(II) oxide nanoparticles, chitosan nanoparticles, calcium phosphate or fluoride nanoparticles, and nanodiamonds can be used in dental therapy and endodontics. Composites with nanoparticles of hydroxyapatite and bioactive glass proved to be of low efficiency for application in these fields. The materials applicable in orthodontics include nanodiamonds, silver nanoparticles, titanium(IV) and zinc(II) oxide nanoparticles, bioactive glass, and yttrium(III) fluoride nanoparticles. Composites of silver nanoparticles and zinc(II) oxide nanoparticles are used in periodontics, and nanodiamonds and silver, chitosan, and titanium(IV) oxide nanoparticles are employed in dental implantology and dental prosthetics. Composites based on titanium(IV) oxide can also be utilized in maxillofacial surgery to manufacture prostheses. Composites with copper(II) oxide nanoparticles and halloysite nanotubes are promising materials in the field of denture prosthetics. Composites with calcium(II) fluoride or phosphate nanoparticles can be used in therapeutic dentistry for tooth restoration.

## 1. Introduction

The terms nanoparticle, nanomaterial, and nanotechnology came into use in the second half of the 20th century and are identified by researchers with innovation and progress in science and industry. Currently, a high-priority area in global science is manufacturing of materials that contain particles comparable in size with molecules, which are called nanoparticles. They have found applications in medical imaging [1], biomedicine [2,3], pharmacology [4], photoelectronics [5] and optoelectronics [6,7], construction [8], photocatalysis [9], as components of heterogeneous catalysts [10,11], as a means of improving the mechanical [12] and thermal properties of polymeric materials [13], sensors [14] and biosensors [15], components of lithium-ion batteries [16], and sorbents [17].

A promising application area of composite nanomaterials is medicine, including dentistry, in which they are used for diagnosis, dental prosthetics, and prevention and treatment of diseases of the oral mucosa and hard tissues of teeth. These materials can reproduce mechanical, physicochemical, and esthetic properties of the hard tissues of teeth and often surpass them in strength characteristics. One more advantage of nanomaterials over traditional composites is that dental restoration can be made with good esthetic characteristics, identical to those of hard tooth tissues, as they possess better optical properties [18].

Decreasing the microbial action in oral cavity organs is an important issue in the prevention and treatment of caries and in restorative dentistry and dental prosthetics. Their large surface, very small size, and high surface energy and charge density allow nanoparticles to interact with the cell membrane, easily penetrate into a pathogen cell, and induce pathogen death [19].

**Figure 1:**
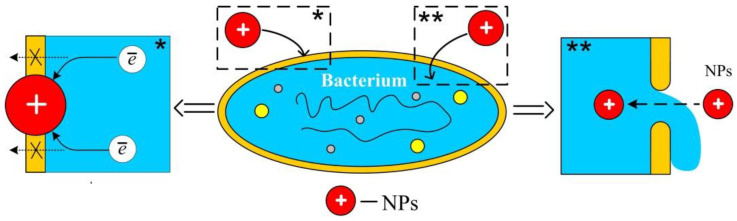
Mechanism of antimicrobial effect of positively charged nanoparticles (using the example of chitosan nanoparticles).

Nanoparticles with a positive charge interact electrostatically with the negatively charged surface of the bacterial cell wall, disrupting the permeability of the cell membrane. In the first case, the respiratory chain of electron transport is blocked (* in Figure 1). In the second, the nanoparticle destroys the membrane, entering the cytoplasm of the bacterial cell, leading to the outflow of intracellular contents and death of the bacterial cell (** in Figure 1).

Previously, an extremely toxic amalgam was used as an antimicrobial dental material [20], which also has poor esthetic properties [21]. Since the 1970s, a less toxic glass ionomer cement (below referred to as GIC) with antimicrobial activity [22], but which has poor mechanical characteristics, has been used. The use of antibiotics leads to antibiotic resistance of microorganisms, as well as a large number of side effects on the part of the digestive system and the central nervous system [23], which limits their use in dentistry.

That is why, in the battle against pathogens, studies devoted to the development of various dental nanocomposites and devoid of most of the above disadvantages become extremely relevant and in demand. For example, studies [24,25] report that nanosized fillers of various classes impart reinforcing, antibacterial, remineralizing, self-curing, radiopaque, and esthetic properties to dental materials. Fauzi et al. [26] developed an esthetic adhesive composite material with antibacterial properties for use with esthetic orthodontic brackets.

**Figure 2:**
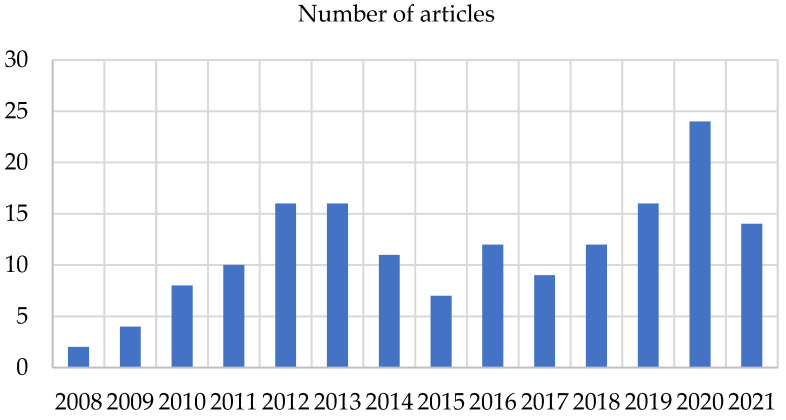
Number of articles on the antimicrobial activity of polymeric nanomaterials for dental applications (PubMed database).

Figure 2 shows the number of publications on polymeric dental nanomaterials with antimicrobial activity. The graph shows that most active development in this area was in 2012, 2013, 2019, and 2020. Until 2008, there were no publications on this topic. 

It should be mentioned that more than 100 reviews published in the period of 2018–2021 are devoted to the use of various types of nanoparticles in dentistry. These publications consider the use of nanoparticles in implantology [27,28], dental therapy [29,30], local drug delivery to treat periodontitis [31], in the treatment of oral cavity cancer [32], and in toothpastes [33]. However, the published reviews do not give detailed descriptions of the antibacterial or antifungal action of dental polymeric nanomaterials and prospects for their application in various fields of dentistry.

The present paper gives an overview of studies published in the period of 2018–2021 that address the antimicrobial properties of nanomaterials related to their use in dentistry. The information on nanoparticles used for dental materials is summarized in Table S1 in the Supplementary Materials.

## 2. Nanoparticles with Antimicrobial Action Used in Dental Materials

A variety of microorganisms are known in dentistry as pathogenic (Supplementary Materials, Table S2), and nanoparticles are active suppressors of many of them (Supplementary Materials, Table S3). Nanoparticles applicable as antimicrobial components include metal, oxide, diamond, glass, and polymeric nanoparticles.

Apart from their antimicrobial action, inorganic nanoparticles are able to improve the integrity of the hybrid layer at the resin–dentin interface [34,35], inhibit enamel demineralization [36] and formation of white spot lesions [37,38] during orthodontic treatment, increase the compressive strength [39,40] and microhardness [41] of restorations based on glass ionomer cement, and reduce polymerization shrinkage of light-curing dental composites [42] and the surface roughness of dentures [43,44] and implants [45] (Supplementary Materials, Table S4).

The inorganic nanoparticles with antimicrobial action studied in recent years include silver, diamond, zinc(II) oxide, titanium(IV) oxide, zirconium(IV) oxide, magnesium(II) oxide, hydroxyapatite, glass, yttrium(III) fluoride, calcium(II) fluoride, and calcium(II) phosphate nanoparticles.

Biocompatible and biodegradable polymeric nanoparticles have shown a high therapeutic potential for controlled drug delivery techniques. The dosage forms based on polymeric nanoparticles penetrate deep into tissues, which increases the drug exposure time and efficiency and minimizes side effects.

Polymeric nanoparticles used in dentistry are subdivided into natural (chitosan, starch, sodium alginate) and synthetic ones (polylactide, poly(lactide-co-glycolide), polyethylene glycol–polylactide). Chitosan holds prospects for use in dentistry as an antimicrobial material.

Here we consider the general characteristics of nanoparticles that have antimicrobial action. 

*Silver nanoparticles* (below referred to as Ag NPs) are spherically shaped biocompatible metal nanoparticles with a controllable size in the range from 3 to 35 nm [18].

Yaqoob et al. [46] discussed in detail chemical, physical, photochemical, and biological methods for the synthesis of silver nanoparticles. It is reported that the most simple, economical, and environmentally friendly method for the synthesis of silver nanoparticles is the biological or green method using plant extracts.

Ag NPs prepared by the reduction of silver nitrate with sodium borohydride or by green synthesis [47,48] show a long-term activity against resistant microorganisms that cause caries, periodontitis, peri-implantitis, inflammation of root canals, and oral candidiasis. However, a tendency to aggregation increases the average size of Ag NPs, which may result in the loss of antibacterial properties. The stability of Ag NPs can be enhanced by using surfactants that contain polar groups and are capable of interacting with surface atoms [49], or by synthesizing Ag NPs in a polymeric matrix [50].

*Nanodiamonds* (below referred to as NDs) are nanocarbon materials characterized by high strength, hardness, optical transparency, low cytotoxicity, and better chemical stability and biocompatibility than metal or metal oxide nanoparticles. The hydroxy, amino, and carboxyl groups present on the NDs’ surface improve their interfacial interactions with polymethyl methacrylate (below PMMA), polyethyl and polybutyl methacrylates, and urethane dimethacrylate resin. The antimicrobial effect of NDs is caused by negatively charged acid anhydride groups present on the surface [51].

*Zinc(II) oxide nanoparticles* (below referred to as ZnO NPs), characterized by biocompatibility, low micro-density, and activity against *S. mutans*, *E. faecalis*, *S. mitis*, *Lactobacillus* spp., *P. gingivalis*, and *A. naeslundii*, have proved to be efficient in endodontic and orthodontic sealers, dental filling materials, and interim dental prostheses [34].

*Titanium(IV) oxide nanoparticles* (below referred to as TiO_2_ NPs) have spherical shape, a smooth surface, and uniform size distribution. TiO_2_ NPs possess useful properties that make them suitable for preparation of dental filling materials, such as chemical stability, biocompatibility, tooth enamel-like color, and a hydrophilic surface. TiO_2_ NPs with a particle size of less than 50 nm possess photoinduced activity and can release free radicals damaging the DNA of *S. mutans* and *S. aureus* bacteria [52].

*Zirconium(IV) oxide nanoparticles* (below referred to as ZrO_2_ NPs) are oval or spherical particles with a surface area of 9 m^2^ g^−1^ and an average size of 40 nm.

ZrO_2_ NPs increase biaxial flexural strength and Vickers microhardness of GIC [41] and increase the tensile and compressive strength and chemical stability of PMMA [53].

*Magnesium oxide(II)nanoparticles* (below referred to as MgO NPs) possessing antibacterial activity are white hygroscopic particles with an average size of 20 nm. MgO NPs are biocompatible, biodegradable, nontoxic, environmentally friendly, and cheaper than Ag NPs. However, like Ag NPs, they are prone to aggregation, which reduces the activity of nanoparticles against bacteria. Aggregation can be prevented by using cellulose, which provides a closer contact of nanoparticles with bacteria and thus bacterial growth can be inhibited by almost 100% [54].

*Hydoxyapatite nanoparticles* (below referred to as nHAps) have a composition and structure similar to those of dental tissue, and, hence, they can be used to coat dentin caries lesions, microcracks in teeth, and dentinal tubules, and to enhance remineralization of demineralized dentin matrix and damaged enamel; this is important for dental therapy, implantology, tissue engineering, and treatment of hypersensitivity.

nHAps also stimulate proliferation, adhesion, and differentiation of mouse odontoblast-like MDPC-23 cells [55]; they enhance the biocompatibility of silver coatings of titanium implants with human primary osteoblasts [56].

*Bioactive glassnanoparticles* (below referred to as BGN) consist of silicon dioxide, calcium oxide, sodium oxide, and phosphorus(V) oxide. In the oral cavity, this glass can release Ca^2+^, PO_4_^3−^, and CO_3_^2−^ ions, thus increasing pH of the medium and inactivating bacterial enzymes. They can also form hydroxycarbonate apatite (the product of crystallization of calcium phosphate on the glass surface), which takes part in the occlusion of dentinal tubules and enamel remineralization [57]. Bioactive glass nanoparticles have an irregular morphology and are prone to aggregation.

*Yttrium(III) fluoride nanoparticles* (below referred to as YFN) *and calcium fluoride nanoparticles* (below referred to as nCaF_2_) can affect the mineral layers of teeth, thus enhancing remineralization [58,59]. The average size of YFN is 60–70 nm [58], and the average size of nCaF_2_ is 32 nm [59].

*Calcium phosphate nanoparticles* (below referred to as NCP) are able to continuously release calcium ions and phosphate ions into the oral cavity, providing a remineralizing effect. They are amorphous calcium phosphate with a high surface area of 17.8 m^2^ g^−1^. Such systems are synthesized by spray drying, carried out by spraying an acetic acid solution of calcium carbonate and dicalcium phosphate into a heated chamber. Average particle size is 116 nm [60].

*Copper (II) oxide nanoparticles* (below referred to as CuO NPs) have an average size of 18 nm. For the synthesis of copper oxide nanoparticles, ethanolic solutions of copper acetate and sodium hydroxide are used. They inhibit biofilm growth in soft denture liners in a dose-dependent manner [61].

*Chitosan nanoparticles* (below referred to as Cs NPs) are obtained by coagulation of the polymer from solutions of various concentrations in a treatment with acetic acid and cross-linking with sodium tripolyphosphate to form a polymer complex. The nanoparticles of chitosan—which is a biologically active, biocompatible, and biodegradable polymer—are used as carriers for targeted delivery of drugs such as doxycycline [62], amoxicillin and clavulanic acid [63], and simvastatin [64] to damaged dental tissue, which ensure prolonged drug release and thus decrease the therapeutic dose.

*Halloysite nanotubes* (below referred to as HNTs) are biocompatible aluminosilicate layers of a tubular structure with a diameter of several tens of nanometers and a length of approximately 200 nm [65].

## 3. Dental Therapy, Endodontics, and Periodontics

In dental therapy, nanomaterials are used as chemically curing, light-curing, and self-curing polymeric composites, glass-ionomer cements, insulating coatings, adhesives, and fissure sealants, while in endodontics such materials are used as root canal sealers.

### 3.1. Materials Containing Silver Nanoparticles

In dental therapy, composites with Ag NPs are used in dental restorative materials based on zirconia and GIC and used to treat carious lesions caused by *S. mutans*, *S. salivarius*, *L. acidophilus*, *C. albicans*, and *C. glabrata.*

A group of Polish researchers found that the addition of Ag NPs into the glass-ionomer cement Ketac Molar EasyMix, and adhesive systems Clearfil SE Bond and OptiBond Solo Plus, enhances the inhibition of growth of Gram-positive bacteria such as *S. mutans*, *S. salivarius*, and *L. acidophilus* after 48 h of observation [66].

Oh et al. [67] evaluated the activity against *S. mutans* of a filling material used for dental restoration based on zirconium dioxide coated by a glass-ceramic powder with addition of 5, 10, 15, and 20 wt. % Ag NPs or NaF and found the following:

(1) The addition of 10 wt. % and 20 wt. % Ag NPs decreases bacterial activity by 11.8% and 15.4%, respectively (Table 1);
(2) The addition of 5–15 wt. % NaF decreases the number of bacteria by 65%, but when the NaF content increases to 20 wt. %, the number of bacteria grows by 29% (Table 1);
(3) Irrespective of Ag NPs and NaF content, the viability of L929 mouse fibroblast cells exceeds 70% for all samples, which attests to the safety of the composites.

However, the authors gave no explanation for the higher rate of inhibition of the cells growth of *S.* *mutans* by NaF than by Ag NPs.

The mechanical endodontic treatment leaves significant areas of *E. faecalis* biofilm and necrotic tissues, which cause apical periodontitis. This problem can be solved by using Ag NP-based dual-cure adhesives, self-etch adhesive systems possessing long-term antibacterial action. Baras et al. [50] developed a dual-cure endodontic sealer based on dimethylamino hexadecyl methacrylate, a glass filler, and BTH resin (a mixture of bisphenol A glycidyl dimethacrylate, triethylene glycol dimethacrylate, 2-hydroxyethyl methacrylate, and methacryloyl oxyethyl phthalate) with addition of 0.15 wt. % Ag NPs. As a result, the concentration of *E. faecalis* bacterial cells was reduced in comparison with commercial AH-Plus sealer from 10^7.4^ to 10^4.7^ CFU mL^−1^, thus preventing the secondary infection of the canals.

Modification of an acrylate self-etch adhesive system with an ethanol dispersion of Ag NPs resulted in increasing diameter of the inhibition zone of *S. mutans* on an agar plate from 11.6 mm to 13.8 mm. However, upon addition of the Ag NPs’ dispersion to an adhesive, the curing degree decreased from 50% to 26% since ethanol diluted the adhesive system [68].

The use of orthodontic retainers that control the position of front teeth after orthodontic treatment is often accompanied by an increasing area of bacterial biofilm. This elevates the risk of periodontal inflammation. To prevent biofilm growth, the addition of Ag NPs to light-curing composite for orthodontic retainers was proposed [69]. A comparison of the activities of composites with Ag NPs (1 wt. %) and without Ag NPs against *T. denticola* showed bacterial viability two orders of magnitude lower for Ag NP- containing samples than for samples without nanoparticles (6 · 10^4^ CFU μL^−1^ and 3 · 10^6^ CFU μL^−1^, respectively).

It is known that periodontal dressing should protect the wound surface after periodontal surgery and facilitate fast healing of the wound tissue. Therefore, Ag NPs were added to increase the efficiency of the polyvinyl alcohol-based dressing, and characteristics of post-surgery periodontal inflammation in rats were estimated using the ^99m^Tc-ciprofloxacin radiopharmaceutical. Despite the fact that the content of the ^99m^Tc-ciprofloxacin marker at the site of dressing with Ag NPs increased after 2 days due to the body’s response to the foreign material, after 4 days the proportion of inflammation decreased [70].

Thus, Ag NPs are promising additives to polymeric materials used in dentistry for periodontics. This is due, first of all, to their anti-inflammatory and wound-healing activity. In addition, silver nanoparticles stabilized by biopolymers have high biocompatibility with human gingival fibroblasts [71].

### 3.2. Materials Containing Zinc Oxide Nanoparticles

**Figure 3:**
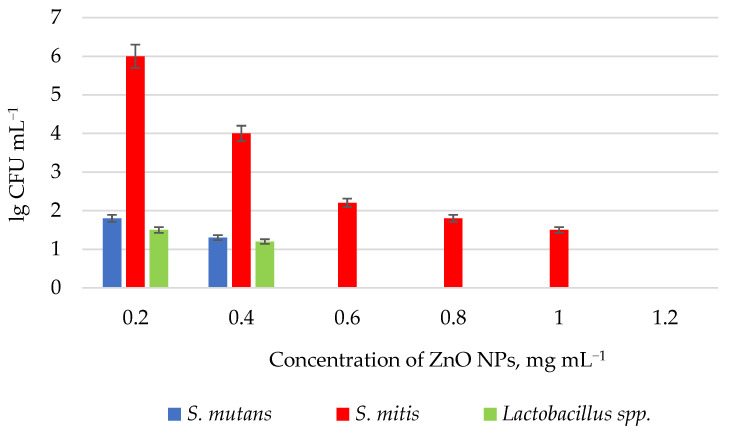
Effects of ZnO NPs (lg CFU mL^−1^) on the growth of *S. mutans* (blue), *S. mitis* (red), and *Lactobacillus* spp. (green) in the thioglycolate broth after 18 h at 37 °C under microaerophilic conditions [72].

ZnO NPs proved to be efficient against anaerobic Gram-positive *S. mutans*, *S. mitis*, and *Lactobacillus* spp. strains under microaerophilic conditions, which mimic a carious cavity [72]. As shown in Figure 3, minimum inhibitory concentration (MIC) values for ZnO NPs were 1.2 mg mL^−1^ for *S. mitis* and 0.6 mg mL^−1^ for *S. mutans* and *Lactobacillus* spp. In addition, a slight bactericidal effect was observed at a concentration of only 0.2 mg mL^−1^. The obtained material was meant for the use in resin filler materials.

Angel Villegas et al. [72] also found that ZnO NPs in Icon methacrylate resin penetrate to a depth of up to 1020 μm from the tooth surface, which indicates a good infiltration ability of the nanomaterial.

The antimicrobial effect of ZnO NPs allowing for overcoming antibiotic resistance of *S. mutans*, *E. faecalis*, *L. fermentum*, and *C. albicans* was detected by the disk diffusion test and microdilution method [73]. In control groups, the antibiotics gentamicin and ampicillin were used for the abovementioned bacteria and fluconazole was used for the *C. albicans* fungus. The results showed an increase in the antimicrobial activity as the particle size decreased from 140 nm to 20 nm. The greatest inhibition zones against *S. mutans* were observed for 20 nm and 40 nm ZnO NPs, whereas 140 nm ZnO NPs formed the greatest inhibition zones against *S. mutans* and *E. faecalis*. The inhibition zones of *C. albicans* were the smallest for all three sizes of ZnO NPs (Table 2). In this regard, based on the data, nanoparticles were used in root canal polymer sealers. It was found that the diameters of inhibition zones against *P. gingivalis* and *A. naeslundii* were greater for ZnO NPs (18.09 mm and 12.05 mm) than for commercial light-curing AH Plus A sealer (9 mm) [74].

The antimicrobial activity of nanoparticles based on ZnO NPs and ZnO NPs encapsulated in chitosan against microorganisms *B. subtilis*, *S. aureus*, *S. hemoliticus*, *P. aeruginosa*, *K. pneumoniae*, *E. coli* was studied (Table 3). *S. mutans* and *L. acidophilus* biofilm inhibition was tested using 3M ESPE Adper™ Single Bond Adhesive dental adhesive disks containing nanoparticles [75].

It should be mentioned that ZnO NPs with chitosan showed stronger antimicrobial activity than without the polymer. According to the authors, this is due to the synergy of the bactericidal activity of ZnO NPs and chitosan.

As for composites based on modified adhesives, the authors [75] describe their anti- caries effect from two positions: (1) preventing the development of recurrent caries on teeth previously treated for it by affecting the structural elements of dentin; (2) reduction in the number of cariogenic bacteria such as *S. mutans* and *L. acidophilus*.

According to the results obtained (Table 4), adhesives containing ZnO NPs and chitosan ZnO NPs reduced the number of cariogenic bacteria *S. mutans* and *L. acidophilus* by four orders in comparison to the control group (adhesive without nanoparticles).

However, the contribution of *L. acidophilus* to the pathogenesis of carious disease is ambiguous, which is explained by their antagonistic effect on true cariogenic strains of microorganisms (*S. mutans*, *S. sanguinis*, *S. salivarius*) on the one hand, and the production of 2-hydroxypropanoic acid, which has a demineralizing effect on inorganic part of enamel and dentin, on the other hand.

### 3.3. Materials Containing Titanium(IV) Oxide Nanoparticles

A comparison of the antibacterial activity of GICs containing antibiotics and TiO_2_ NPs [40] showed that the inhibition zone of TiO_2_ NPs containing GIC against *S. mutans* was 21.2 mm, that of cetylpyridinium chloride-containing sample was 18.3 mm, and that of ampicillin-containing sample was 31.2 mm. Meanwhile, ampicillin particles deteriorate the interaction between glass particles and liquid cement and, hence, decrease the compressive strength of GIC. Conversely, TiO_2_ NPs fill the voids between glass particles and thus increase the compressive strength of GIC from 140 MPa to 173 MPa.

The antibacterial activity against *S. mutans* [76] was also evaluated for an acrylic dental composite resin, Filtek Z350 XT, filled with TiO_2_ NPs. The direct contact assay showed that increase in the content of nanoparticles results in decreasing bacterial growth. The incorporation of 2% TiO_2_ NPs into the resin reduced the bacterial concentration in the culture broth (BHI + 1% saccharose) by 75% without deterioration of mechanical or physicochemical properties.

Thus, TiO_2_ NPs can be used as antibacterial fillers for materials meant for tooth restoration.

Florez et al. [52] investigated the OptiBond Solo Plus dental acrylic adhesives containing TiO_2_ NPs for activity against *S. mutans* biofilms. Determination of viable bacterial counts using bioluminescence assay showed that the antibacterial properties of the samples increase with increasing content of TiO_2_ NPs, irrespective of the time of bacterial growth (3 to 24 h). The authors also showed that nitrogen-doped TiO_2_ NPs are photoactive and their antibacterial properties increase upon long-term (24 h) irradiation with light. However, it is unclear how long-term irradiation of this adhesive can be carried out in an oral cavity.

### 3.4. Materials Containing Magnesium(II) Oxide Nanoparticles

In a study of the action of GIC modified by MgO NPs on *S. mutans* and *S. sobrinus*, the agar diffusion test demonstrated that GICs containing up to 1 wt. % MgO NPs do not suppress bacterial growth [77,78]. As the concentration of MgO NPs increases, the diameter of inhibition zones increases and reaches 9 mm for 10 wt. % MgO NPs.

The biocompatibility of MgO NPs makes them perfect candidates for clinical use in dentistry as parts of polymeric filling and restoration materials. However, as indicated above, the antibacterial activity is manifested only when the nanoparticle content in the composites is higher than 1 wt. %.

### 3.5. Materials Containing Hydroxyapatite Nanoparticles

The introduction of nHAps in an amount of 10 wt. % to 30 wt. % in acrylic binder based on bisphenol A glycidyl dimethacrylate and triethylene glycol dimethacrylate promotes remineralization of enamel affected by *S. mutans* biofilm [79]. For 98 days of observation, it was found that with an increase in the concentration of nanoparticles in the composite, the released amount of calcium ions and phosphate ions also increased. It is also reported that at potentially cariogenic pH = 4, more calcium and phosphate ions are released than at pH = 7 (oral pH).

However, it should be noted that with an increase in the content of nHAps in composites, their bending strength decreased. The authors explain this fact by the poor interaction between the organic matrix and mineral nHAps. A statistically significant decrease in the translucency of the composites was also observed with an increase in the content of nHAps from 20 wt. % to 30 wt. %. Unfortunately, the authors do not provide an explanation for this fact.

### 3.6. Materials Containing Bioactive Glass Nanoparticles

Al-Bakhash et al. [80] estimated the activity of an epoxy resin-based dental sealer, Dentsply Maillefer, modified by various nanofillers: hydoxyapatite, fluorohydroxyapatite, and BGN against Gram-positive *E. faecalis* and *S. mitis* bacteria. The most pronounced antimicrobial properties were observed for sealers doped with fluorohydroxyapatite nanoparticles, since Gram-positive bacteria with negatively charged peptidoglycans are more susceptible to hydroxyapatite than to bioactive glass (CFU mL^−1^ of *E. faecalis* and *S. mitis* decreased by 15% and 17%, while in the case of BGN, the decrease was only 2% and 4%). Thus, bioactive glass is barely useful for dental therapy.

### 3.7. Materials Containing Chitosan Nanoparticles

A comparison of the antibacterial properties against *S. mutans* for three composites based on Cs NPs (83 nm size), Cs NPs/ZnO NPs (186 nm size), and ZnO NPs (38 nm size) demonstrated that the introduction of ZnO NPs into the acrylic composite resin Filtek Z250 Universal Restorative provides an antibacterial effect, which is retained for up to 12 weeks, while for chitosan-containing composites it is retained only for 2 weeks. The inhibition zone was also larger for microhybrid composites containing ZnO NPs than for composites based on Cs NPs (Table 5). This may be due to the smaller size and low tendency for aggregation inherent in zinc oxide nanoparticles and, hence, to their greater surface publications.

Thus, according to published data, polymeric materials filled with Ag NPs and TiO_2_ NPs are most promising for the use in dental therapy and endodontics, first of all, for the treatment of primary and secondary caries lesions. This is due to the fact that silver nanoparticles and titanium(IV) oxide nanoparticles can simultaneously improve the antimicrobial and mechanical properties of dental material. Ag NPs and TiO_2_ NPs occupy voids in the GIC, acting as additional contact points between the binder and glass particles. This increases the compressive strength [39,81], flexural strength, and Vickers microhardness of the composites and the micro-shear bond strength to dentin [82].

### 3.8. Materials Containing Calcium Phosphate Orcalcium Fluoride Nanoparticles

Most modern composite dental materials are formed in the process of photopolymerization of low molecular weight binders. In this regard, there is a problem of shrinkage, which often leads to damage to fillings and restorations in the form of microcracks, which contributes to their colonization by microorganisms. A study was conducted aimed at reducing the shrinkage of composites [83]. The composite included urethane dimethacrylate, thriethylene glycol divinylbenzyl ether, and dimethylaminohexadecyl methacrylate. NCP (20 wt. %) and silanized barium boroaluminosilicate glass particles (43 wt. %) were used as fillers.

The resulting material had a 40% lower polymerization stress compared to the commercial Heliomolar composite (Ivoclar, Ontario, Canada). In addition, the developed composite material protected tooth enamel from demineralization caused by *S. mutans* biofilm by 5.17 ± 0.48 mmol L^−1^. The cytotoxicity of the composite material in relation to the human gingival fibroblast cell was similar to that of the composite based on bisphenol A glycidyl dimethacrylate, which indicated that the developed material is suitable for clinical use.

Also of interest are composite materials containing fluoride anions that release them into the environment surrounding the material. The authors of [59] fabricated a composite based on acrylic resin containing 15 wt. % nCaF_2_ as filler and 3 wt. % dimethylaminohexadecyl methacrylate. The CFU values of biofilm grown on composite disks decreased by four orders of magnitude compared to the commercial composite Heliomolar (Ivoclar Vivadent, Mississauga, ON, Canada). The release of fluoride ions after 70 days of observation was 0.20 ± 0.03 mmol L^−1^, calcium ions 0.18 ± 0.005 mmol L^−1^, while for the commercial composite the release of calcium and fluoride ions was close to zero. The developed material also significantly (by 60%) reduced the production of lactic acid by bacteria compared to the commercial composite.

However, the authors of works [59,83] did not take into account the remineralizing effect of saliva and used only one type of bacteria, *S. mutans*.

Summarizing, we can conclude that materials filled with zinc oxide and chitosan nanoparticles, as well as nanoparticles of calcium phosphate and fluoride, are highly active against the main caries bacteria (*S. mutans*). Zinc oxide nanoparticles are also active against bacteria causing periodontitis and apical periodontitis (*P. gingivalis*, *A. naeslundii*) and can be used as additives to fillers and sealers for filling root canals and for treatment of periodontitis.

Restorative materials filled with magnesium oxide, bioactive glass, and hydroxyapatite nanoparticles have low efficacy against pathogens and are fluoride inapplicable as antimicrobial additives.

## 4. Orthodontics

In orthodontics, nanoparticles are added to orthodontic adhesives and acrylic resins and are used as coatings for orthodontic appliances, in particular, orthodontic brackets.

Orthodontic appliances such as metallic and esthetic braces, rings, arcs, and bands complicate oral hygiene and create favorable conditions for the growth of *S. mutans*, *S. aureus*, *S. sobrinus*, *S. sanguis*, *P. gingivalis*, *E. coli*, and *L. acidophilus* biofilms, which increases the risk of tooth surface lesions.

In the field of orthodontics, most prospective are materials containing silver, titanium(IV) oxide, zinc(II) oxide, bioactive glass, and yttrium(III) fluoride nanoparticles and nanodiamonds.

### 4.1. Materials Containing Silver Nanoparticles

Orthodontic adhesives containing Ag NPs decrease bacterial adhesion at the brace/enamel interface.

The adhesive composite materials meant for attachment of orthodontic brackets should also possess antimicrobial action in order to prevent the formation of white spots. Lee et al. [84] tested an antimicrobial resin based on the *Transbond XT* primer with the addition of Ag NPs. The antibacterial activity of this resin was evaluated in vitro against two oral pathogens, *S. mutans* and *S. sobrinus*. In both cases, the percentages of viable bacterial cells considerably decreased.

A comparison of the antibacterial activities of this resin against *S. mutans* and *L. acidophilus* indicated that the diameter of the inhibition zones against *S. mutans* growth was greater than that against *L. acidophilus* growth [85].

The authors of [86] studied the antimicrobial effect of composite resin disks based on acrylic composite resin Flow Tain (Reliance, Houston, TX, USA), containing 1 wt. %, 2 wt. %, and 5 wt. % Ag NPs, against the bacteria *S. mutans*, *S. sanguis*, and *L. acidophilus*. The results obtained are presented in Table 6.

According to the data obtained, composites containing AgNPs reduce the number of colonies of microorganisms in a dose-dependent manner. The largest decrease in CFU was observed for the biofilm of oral bacteria *S. sanguis* compared to *S. mutans* and *L. acidophilus.*

However, the zone of inhibition was observed only for composites with the highest content of nanoparticles, equal to 5 wt. %. The authors do not provide an explanation for this fact.

The resulting composites, according to the authors, after clinical trials can be used in orthodontics as orthodontic appliances.

### 4.2. Materials Containing Titanium(IV) Oxide Nanoparticles

In a comparison of antibacterial activities against *S. mutans* in three groups of Transbond XT acrylate composites modified with Ag NPs, ZnO NPs, and TiO_2_ NPs, the viable bacterial count decreased in all cases. Nevertheless, the antibacterial activity was higher for the TiO_2_ NP-containing group than for the Ag NPs and ZnO NP-containing groups, which is due to smaller size of TiO_2_ NPs (25 nm) compared with Ag NPs (80 nm) and ZnO NPs (50 nm) [87].

The antibacterial properties of composite disks based on a thriethylene glycol dimethacrylate, diurethane dimethacrylate resin mixture (50:50 wt./wt.) and nitrogen-doped TiO_2_ NPs were studied in relation to cariogenic bacteria *S. mutans* [26]. The nanoparticles were doped with nitrogen to prevent discoloration of the resin composite containing the nanoparticles after exposure to visible light. The antibacterial effect was assessed by the metabolic activity of bacterial cells under illumination conditions (indicator—tetrazolium salt). A decrease in absorption was observed with the addition of TiO_2_ NPs, and with increasing concentration of nanoparticles, the decrease in absorption increased (Table 7). As can be seen from Table 7, the surface treatment of the composite with both polishing and plasma contributed to the improvement in antibacterial action compared to untreated composites.

In the future, the authors plan to investigate the effect of other microorganisms on the inhibitory effect of TiO_2_ NPs doped with nitrogen.

### 4.3. Materials Containing Nanodiamond

It was shown by Mangal et al. [88] that composites based on Ortho-Jet orthodontic acrylic resin meant for the manufacture and repair of orthodontic appliances, containing 0.1, 0.3, and 0.5 wt. % of ND powder, are active against *C. albicans* fungi. The CFU count decreased almost to zero upon the addition of only 0.1 wt. % NDs to the resin. The biofilm thickness and weight decreased for all concentrations of NDs. However, further studies are required to establish the mechanism of interaction of composites with the microbes. It is also noteworthy that the addition of NDs deteriorated the optical properties of the composite.

In work [89], the antibacterial and tribological properties of a composite based on PMMA containing 0.1 wt. % ND powder were studied by Mangal et al. For this, samples were obtained by 3D printing and studied in vitro. A polymer without nanoparticles served as control sample.

The severity of the antibacterial effect of the composite was assessed by the resistance to the formation of biofilms based on *S. mutans* on these materials for 48 h. It was found that the biofilm thickness decreased from 200 μm (control group) to 150 μm (nanocomposite), while biofilm biomass decreased from 120 μm^3^ μm^−2^ (control group) to 30 μm^3^ μm^−2^ (composite).

In addition, composite increased Vickers microhardness and wear resistance, as well as reduced the coefficient of friction, compared with the control group. The pronounced antibacterial effect and good performance characteristics of diamond composites allow them to be recommended for use in orthodontics for the manufacture of orthodontic appliances.

### 4.4. Materials Containing Zinc(II) Oxide Nanocomposites

Pourhajibagher et al. [90] evaluated the antimicrobial properties against *S. mutans*, *S. sobrinus*, and *L. acidophilus* for the Transbond XT acrylic orthodontic adhesive containing cationic curcumin-doped ZnO NPs. The results, summarized in Table 8, indicate than the samples retained antimicrobial properties after 180 days of observation.

### 4.5. Materials Containing Bioactive Glass Nanoparticles

Nam et al. [36] modified the low-viscosity acrylic orthodontic bonding resin Transbond Supreme LV with BGN particles. Samples with 3 wt. % and 5 wt. % BGN were evaluated for the antibacterial activity against Gram-positive *S. mutans* bacteria.

For both concentrations of nanoparticles, the antibacterial activity against *S. mutans* after 48 h of culturing of BGN samples in the BHI broth was higher than that for unfilled bonding resin.

### 4.6. Materials Containing Yttrium(III) Fluoride Nanoparticles

Yttrium(III) fluoride nanoparticles can also be used in orthodontics as a component of orthodontic polymeric adhesive. Asiry et al. [58] evaluated the adhesive strength and antibacterial effect of Transbond XT acrylic orthodontic composite resins mixed with yttrium fluoride nanoparticles, with an average particle size of 60–70 nm. The presence of YFN in the resin in concentration of 1 wt. % induced a considerable antibacterial effect against *S. mutans*, as indicated by CFU decrease from 75.85 to 2.24. There was no statistically significant decrease in the bracket–enamel adhesive strength (11.61 MPa in the control group and 11.44 MPa in the test group). The antibacterial action of YFN was attributed [57] to the formation of metal fluoride complexes with bacterial peroxidases. It is noteworthy that an increase in the nanoparticle concentration to 2% decreased the antibacterial activity; however, the authors did not give an explanation for this fact.

An advantage of YFN over all other nanoparticles considered above is the presence of remineralization activity apart from the antibacterial activity.

However, compared to composites based on YFN, the introduction of zinc(II) oxide composites into orthodontic acrylic resins improves both antimicrobial and mechanical properties of orthodontic appliances, such as flexural strength and Vickers hardness [88]. This makes them most appropriate for orthodontic dentistry applications.

It is worth noting that most of the publications considered in this section address the antimicrobial activity against only one or two types of microorganisms; meanwhile, the oral microflora is quite diverse.

## 5. Dental Implantology and Dental Prosthetics

Modification of the surface of dental implants with nanoparticles reduces the probability of post-implantation infection owing to their antimicrobial action.

### 5.1. Materials Containing Silver Nanoparticles

After dental implantation or prosthetics, an important task is to prevent *C. albicans* c and *C. glabrata* fungal infections in order to avoid repeated surgery because of infection. Silver NPs exhibit higher antimicrobial activity than antifungal drugs such as fluconazole, griseofulvin, itraconazole, and miconazole; therefore, silver nanoparticles are used to modify heat-curing and self-curing acrylic dental composites.

Since dental prostheses made of PMMA are actively colonized by fungi, De Matteis et al. [91] attempted to control fungal infection by adding Ag NPs/sodium citrate of 20 nm diameter to the PMMA-based Paladon 65 material. The negatively charged Ag NPs/sodium citrate particles decreased roughness, porosity, and hydrophobicity of the PMMA surface, thus decreasing adhesion and colonization of *C. albicans* on the polymer.

The metabolic activity of *C. glabrata* was studied in the presence of composites based on Lucitone 550 acrylic resin containing Ag NPs/sodium citrate [92]. It was found that 0.5 to 5 vol. % nanoparticle concentrations in the composite resulted in the formation of aggregates. However, the authors noted that a decrease in the Ag NPs concentration to 0.05 vol. % decreases the metabolic activity of *C. glabrata* biofilms by 43%.

### 5.2. Materials Containing Chitosan Nanoparticles

In order to prevent complications of dental prosthetics such as stomatitis and chronic atrophic candidiasis, it was proposed to deposit a tissue acrylic conditioner containing 40 nm to 100 nm Cs NPs on the complete denture surface [93]. Samples containing 2.5 wt. % to 10 wt. % Cs NPs completely inhibited the growth of *C. albicans* fungi and *S. mutans*, *P. aeruginosa*, and *E. faecalis* bacteria after incubation for 24 h. The inhibitory action of Cs NPs was attributed to the interaction of chitosan, which is a polycation, with anionic components of the cell walls of microorganisms [93].

### 5.3. Materials Containing Nanodiamonds

Fouda et al. [51] estimated the adhesion of *C. albicans*, the main causative agent of the denture stomatitis, to the cured Major Base 20 acrylic resin depending on the content of NDs (0.5–1.5 wt. %). The lowest CFU count (~290 per μL of the resin) was observed for 1% NDs. For the control group, this count was ~1300 per μL of the resin. In turn, the addition of 0.5 wt. % NDs to the resin enhanced the mechanical properties of the composite and decreased the surface roughness [43]. The decrease in roughness deteriorated the microbial adhesion to the denture and improved the esthetic quality of the denture and patients’ comfort.

Acrylic resins with quaternized nanodiamonds (below QNDs) can markedly decrease the number of viable *S. mutans* bacterial cells. After testing, a non-modified polymer disk was mainly covered with viable bacteria, while in case of QND-containing resins, both viable and dead cells were detected on the disk. Furthermore, the area of viable cells decreased with increasing QND concentration. When 1.0 wt. % and 1.5 wt. % QNDs was introduced into the resin, the number of viable bacteria on the disks decreased to 37.9% and 25.3%, respectively. These QND-containing resins are intended for the manufacture of tooth dentures [94].

### 5.4. Materials Containing Zinc(II) Oxide Nanoparticles

The antifungal properties of tissue conditioners such as GC Soft-liner, based on plasticized polyether methacrylate and containing various amounts of ZnO NPs, were evaluated [95]. It is reported that air conditioners containing 15 wt. % ZnO NPs reduced by an order of magnitude the number of *C. albicans* fungi cells compared with the control group after 7 and 14 days of observation. However, air conditioners containing 5 wt. % and 10 wt. % ZnO NPs did not show a statistically significant reduction in the number of *C. albicans* compared to the control group. The authors explain this fact by the possible leaching of ZnO NPs due to the absence of bonds between nanoparticles and the polymer matrix of the air conditioner. In the future, the authors plan to investigate the cytotoxicity of the resulting materials.

### 5.5. Materials Containing Titanium(IV) Oxide Nanoparticles

In the work of Cascione et al. [96], to solve the problem of adhesion of microorganisms to the surface of dentures based on PMMA and the low strength of such dentures, two composites were developed and studied based on PMMA and TiO_2_ NPs, and the same polymer with HNTs.

**Figure 4:**
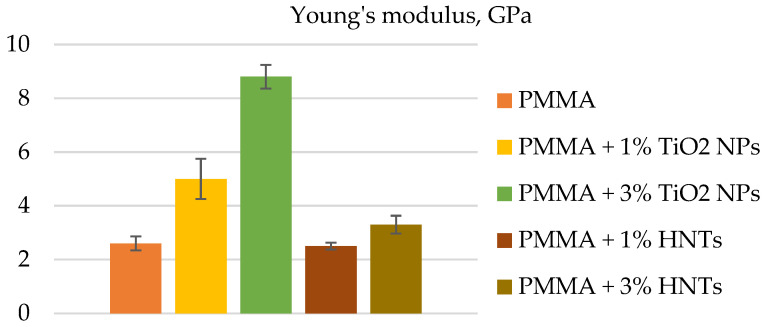
The mean value and its standard deviation of Young’s modulus, evaluated for control (PMMA), PMMA + 1% TiO_2_ NPs, PMMA + 3% TiO_2_ NPs, PMMA + 1% HNTs, and PMMA + 3% HNTs, are represented. Reported results were considered statistically significant respect to control (PMMA) for a *p*-value < 0.005.

Material properties were assessed in terms of physical parameters such as Young’s modulus, roughness, and wettability, and the reduction in the concentration of the diploid fungus *C. albicans* was evaluated. Both types of material showed an improvement in these characteristics in comparison with unmodified composites. The improvement in properties correlated with the concentration of added nanoparticles. At the same time, the authors of [96] found that materials containing TiO_2_ NPs had higher Young’s modulus and, consequently, stiffness, in comparison with materials containing HNTs (Figure 4).

**Figure 5:**
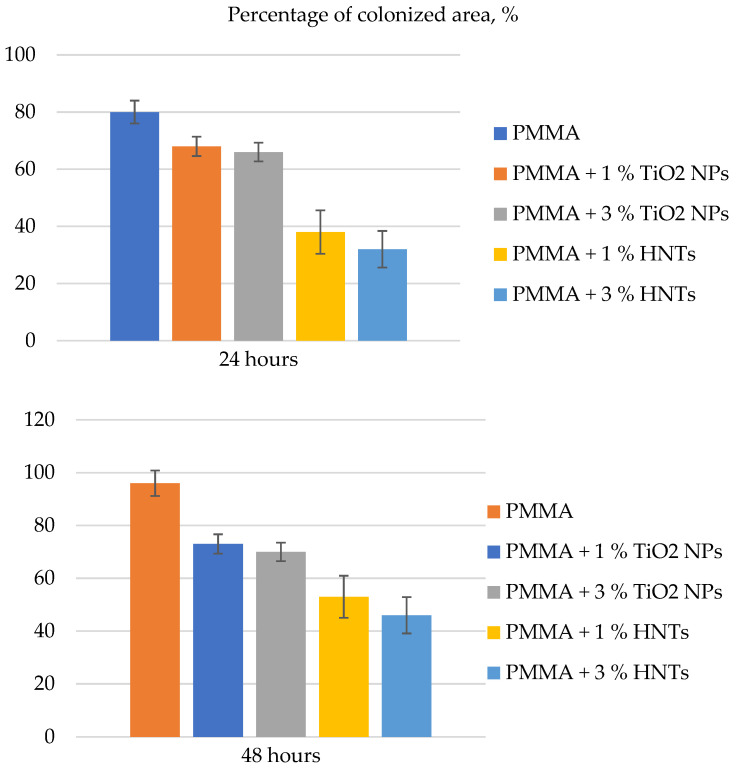
Histograms reporting the colonization assay measurements of *C. albicans* on (PMMA) and different PMMA-based substrates. The colonized area was expressed as a percentage rate representing the area covered by *C. albicans* in respect to the entire surface at two time points (24 h and 48 h). Reported results were calculated as average ± SD for three different areas of each sample, and the values were considered statistically significant in respect to control (PMMA at corresponding time point) for a *p*-value < 0.005.

For *C. albicans*, it was found that composites containing HNTs reduced the rate of colonization by diploid fungi significantly more than materials containing TiO_2_ NPs (Figure 5). However, the authors did not explain this fact.

### 5.6. Materials Containing Copper(II) Oxide Nanoparticles

In a study of the antibiofilm activity of the self-cured acrylic-based GC Soft-liner containing CuO NPs in relation to the *C. albicans* biofilm, it was found that the material inhibits the growth of the *C. albicans* biofilm in a dose-dependent manner (in particular, up to 75% at a concentration of CuO NPs 500 μg mL^−1^) [61].

A comparison was also made of the antimicrobial activity of PMMA-based denture composites containing CuO NPs (2.5 wt. % and 7.5 wt. %) and TiO_2_ NPs (2.5 wt. % and 7.5 wt. %) against fungi of the genus *Candida*—*C. albicans*, *C. dubliniensis*— and bacteria of the genus *Streptococcus (S. mutans*, *S. sobrinus*, *S. salivarius*, *S. sanguis)* [97].

All samples had antimicrobial activity against *S. salivarius*, *S. sanguis*, and *C. dubliniensis*. However, only samples containing 2.5 wt. % and 7.5 wt. % CuO NPs were active against *S. mutans* bacteria. In relation to bacteria *S. sobrinus*, samples with 2.5 wt. % and 7.5 wt. % of TiO_2_ NPs and with 7.5 wt. % of CuO NPs had activity, and in relation to fungi *C. albicans*, only samples with 7.5 wt. % of TiO_2_ NPs showed activity.

Unfortunately, the authors of this work studied composites with only two concentrations of CuO NPs and TiO_2_ NPs.

Summarizing the above, we can conclude that materials containing silver, chitosan, titanium(IV) oxide and copper(II) oxide nanoparticles, halloysite nanotubes, and nanodiamonds have activity against fungi of the genus *Candida*, the main pathogens in the field of dental implantology and dental prosthetics. Materials based on zinc(II) oxide nanoparticles are effective against *C. albicans* only if their content in the composite is high (more than 15 wt. %).

## 6. Maxillofacial Surgery

The introduction of nanofillers into a polymer matrix for the manufacture of maxillofacial prostheses is aimed at improving the physicomechanical properties of prostheses and increasing the compressive strength, flexural strength, and microhardness of materials. However, some of them also possess a high antimicrobial activity, which is important as well.

### 6.1. Materials Containing Titanium(IV) Oxide Nanoparticles

A fundamental goal of maxillofacial prostheses is the restoration of the lost esthetic appearance of the upper and lower jaws. Silicone elastomers filled with TiO_2_ NPs act as an ideal impression material for the elimination of maxillofacial defects owing to the antibacterial properties of TiO_2_ NPs against *S. aureus* and *E. coli*. For example, Salih et al. [98] reported a 2.5-fold increase in the antibacterial activity of a material based on Versiltal RTV silicone elastomer and PMMA with the addition of 0.1 wt. % TiO_2_ NPs.

### 6.2. Materials Containing Bioactive Glass Nanoparticles

Composites based on zein and silver-doped BGN were proposed for jaw bone tissue engineering. The antibacterial properties of the composites against *E. coli* and *S. aureus* were studied in vitro. The experiments were carried out [99] using porous zein/BGN and silver-doped BGN 3D scaffolds. It was noted that the scaffolds containing BGN without silver exhibited no antibacterial activity, unlike the scaffolds containing silver-doped BGN, as indicated by the absence of inhibition zones of bacterial growth. However, the authors did not report the diameters of inhibition zones, which is a considerable drawback of the study.

Over the past two years, there were almost no publications devoted to antimicrobial properties of nanomaterials for maxillofacial prostheses. The researchers’ attention was focused on the cytotoxicity [100], physicomechanical properties [101,102], and osseointegration [103] because these materials should have a mechanical strength similar to bone tissue strength and should be nontoxic on contact with biological fluids and living tissues of the human body. Nevertheless, microorganisms can form biofilms on the surface of maxillofacial prostheses, which gives rise to purulent inflammatory processes; therefore, it would be appropriate to study the effect of nanoparticles on antimicrobial characteristics of materials used in this field of dentistry.

## 7. Prospects for the Application of Dental Materials Containing Nanoparticles of Various Classes

The application of polymeric nanomaterials in dentistry may increase the efficiency of treatment of diseases of hard dental, gingival, and periodontal tissues.

Materials containing silver nanoparticles show promise to be used in filling and restorative dental materials in dental therapy, adhesives and sealers in endodontics, acrylic resins for the manufacture of dentures, and in periodontal materials. The broad scope of applications of materials containing silver nanoparticles in dentistry is due to their lack of toxicity, anti-inflammatory action, and ability not only to endow dental materials with antibacterial and antifungal properties but also to improve their mechanical properties.

Zinc oxide nanocomposites can be used in dental therapy, periodontics, and orthodontics.

However, the antibacterial activity of zinc oxide nanoparticles decreases with aging of dental materials [90]. In addition, zinc oxide nanoparticles of any size and concentration exhibit low antifungal activity.

Titanium dioxide nanocomposites are promising restorative materials and composites for the manufacture of orthodontic appliances and maxillofacial prostheses.

Magnesium oxide nanoparticles deteriorate the mechanical properties of filling materials [76]. They have low efficiency against bacteria, while their antifungal properties are unexplored.

Hydroxyapatite nanoparticles show antimicrobial activity only at high concentrations in dental material. Therefore, hydroxyapatite nanoparticles can be used as additives to filler materials in dental therapy only in combination with silver ions [104].

Zirconium dioxide nanoparticles can adversely affect the physical and esthetic properties of dentures, for example, by increasing surface roughness and decreasing the transparency of PMMA [105]. Therefore, zirconium dioxide nanoparticles are of no interest as dental prosthetics.

Bioactive glass nanoparticles can be used as fillers for polymeric orthodontic materials since, apart from their antibacterial effect, they increase the materials’ hardness [36].

Materials filled with chitosan nanoparticles are less promising for dental therapy and endodontics than materials containing inorganic nanoparticles, e.g., zinc oxide nanoparticles, since they are less efficient against microorganisms. Furthermore, chitosan nanoparticles are cytotoxic to normal human dental pulp cells, substantially reducing their proliferation and viability [106].

Materials containing halloysite nanotubes are promising materials for the manufacture of partial and complete removable dentures in the field of dental prosthetics.

However, most studies of dental materials containing nanoparticles have been based on in vitro experiments, while in vivo studies allow working with multi-species biofilms and are considered more accurate than in vitro ones. Therefore, there is growing need of further experiments in vivo for implementation of these materials into clinical practice.

One of the problems that may limit the use and implementation of dental nanomaterials in clinical practice is their potential toxicity to patient tissues during prolonged exposure to the oral cavity. Toxicity is due to the leaching of nanosized fillers from the material and their effect on the cells of surrounding biological tissues. Toxicity and biocompatibility studies are of great importance for the clinical application of dental nanomaterials, especially in the field of dental implantology and dental prosthetics, since implants and prostheses come into contact with living tissues. However, it should be noted that the toxicity and biocompatibility of dental nanomaterials have not been studied in most of the considered works.

## 8. Conclusions

The presented literature review shows the prospects for the use of polymeric dental materials containing nanoparticles of various classes in such areas of dentistry as dental therapy, orthodontics, dental implantology, dental prosthetics, and maxillofacial surgery in terms of their antimicrobial activity. The innovations described in the review set a new vector of development in relation to restorative dental materials and composites, aimed at improving the quality of life of patients.

Researchers are continuously working on nanoparticle-filled dental materials capable of preserving the natural color of hard dental tissue without change over time, antimicrobial action, low cytotoxicity, high (compressive, tensile, and shear) strength, microhardness, impact strength, radiopacity, and color stability, and also high chemical stability. For example, composites filled with hybrid nanoparticles based on phosphazenes containing carboxyl groups [107,108] and methacrylic groups [109] are promising filler materials.

## Figures and Tables

**Table 1:** Bacterial reduction rate for *S. mutans* on the surface of ZrO_2_ disks coated by glass–ceramic powder containing Ag NPs and NaF after 24 h of observation.

Composition	Content, wt. %	Bacterial Reduction Rate, %
Ag	5	0
	10	0
	15	11.8
	20	15.4
NaF	5	4.2
	10	35.3
	15	65.4
	20	29.4

**Table 2:** Diameters of zones of growth inhibition of microorganisms, mm.

Microorganism	20 nm ZnO NPs	40 nm ZnO NPs	140 nm ZnO NPs	Control
*S. mutans*	16 ± 0.00	14 ± 0.00	12.03 ± 0.57	21 ± 0.00
*E. faecalis*	14.33 ± 0.57	13 ± 0.00	12 ± 1.00	26 ± 0.00
*L. fermentum*	10 ± 0.00	9.33 ± 0.57	8.33 ± 0.57	23 ± 0.00
*C. albicans*	7.66 ± 2.08	6 ± 0.00	6 ± 0.00	16 ± 0.00

**Table 3:** Antibacterial activity exhibited by ZnO NPs, chitosan ZnO NPs against Gram-positive and Gram-negative bacterial strains.

Nanoparticles	Microorganism	Zone of Inhibition
ZnO NPs	*B. subtillis*	7 ± 3
	*S. aureus*	8 ± 3
	*S. heamoliticus*	9 ± 3
	*P. aeroginosa*	9 ± 3
	*K. pneumoniae*	10 ± 3
	*E. coli*	10 ± 3
Chitosan ZnO NPs	*B. subtillis*	9 ± 3
	*S. aureus*	10 ± 3
	*S. heamoliticus*	12 ± 3
	*P. aeroginosa*	10 ± 3
	*K. pneumoniae*	13 ± 3
	*E. coli*	12 ± 3

**Table 4:** Anti-cariogenic activity of adhesive disks containing different concentrations of ZnO NPs and chitosan ZnO NPs.

Nanoparticles	Concentration of NPs, wt. %	Days	Mean CFU mL^−1^
ZnO NPs	0 (control)	1	2.05 × 10^5^
		3	3.26 × 10^5^
		7	3.17 × 10^5^
	2	1	5.44 × 10^1^
		3	4.91 × 10^1^
		7	3.81 × 10^1^
	5	1	5.25 × 10^1^
		3	3.88 × 10^1^
		7	2.87 × 10^1^
	10	1	3.61 × 10^1^
		3	3.26 × 10^1^
		7	3.28 × 10^1^
Chitosan ZnO NPs	2	1	2.42 × 10^1^
		3	1.85 × 10^1^
		7	3.19 × 10^1^
	5	1	2.54 × 10^1^
		3	1.41 × 10^1^
		7	2.84 × 10^1^
	10	1	1.46 × 10^1^
		3	1.16 × 10^1^
		7	1.77 × 10^1^

**Table 5:** Width of the inhibition zone against *S. mutans*, mm.

Sample	24 h	2 Weeks	6 Weeks	12 Weeks
Filtek Z250 microhybrid composite resin	6.0 ± 0.0	6.0 ± 0.0	6.0 ± 0.0	6.0 ± 0.0
Resin + ZnO NPs	18.0 ± 0.71	16.0 ± 1.58	14.0 ± 0.71	8.0 ± 0.71
Resin + Cs NPs	18.0 ± 1.87	10.0 ± 1.0	6.0 ± 0.0	6.0 ± 0.0
Resin + Cs NPs/ZnO NPs	15.80 ± 1.48	10.0 ± 0.71	6.0 ± 0.0	6.0 ± 0.0
*p*	<0.001 *	<0.001 *	<0.001 *	<0.001 *

* Statistically significant at *p* ≤ 0.05.

**Table 6:** Results of biofilm inhibition tests for *S. mutans*, *S. sanguis*, and *L. acidophilus* for composite disks containing Ag NPs and control group.

Sample	Microorganism	CFU	CFU mL^−1^ Decrease (%) Compared to the Control Group
Control	*S. mutans*	56,666 ± 30,550	-
	*S. sanguis*	446,666 ± 117,189	-
	*L. acidophilus*	146,666 ± 32,145	-
Composite disks containing 1 wt. % Ag NPs	*S. mutans*	7000 ± 1000	87.64
	*S. sanguis*	8333 ± 1527	98.13
	*L. acidophilus*	27,000 ± 7549	81.59
Composite disks containing 2 wt. % Ag NPs	*S. mutans*	2000 ± 1000	96.47
	*S. sanguis*	2333 ± 1527	99.47
	*L. acidophilus*	13,333 ± 2516	90.9
Composite disks containing 5 wt. % Ag NPs	*S. mutans*	133 ± 57	99.76
	*S. sanguis*	300 ± 100	99.93
	*L. acidophilus*	566 ± 251	99.61

**Table 7:** The mean absorbance observed for resin disk samples with different wt. % powder concentrations (0–9 wt. %), exposed to different surface treatments (unpolished, plasma treated, polished, and polished with plasma treatment) under light conditions.

Surface Treatment	Concentration of Nitrogen-Doped TiO_2_ NPs, wt. %	Absorbance (a.u.)
Untreated	0	0.072 ± 0.002
	1	0.069 ± 0.005
	3	0.066 ± 0.002
	5	0.060 ± 0.004
	7	0.058 ± 0.004
	9	0.056 ± 0.004
Plasma treated	0	0.072 ± 0.002
	1	0.067 ± 0.004
	3	0.066 ± 0.005
	5	0.060 ± 0.004
	7	0.056 ± 0.004
	9	0.055 ± 0.004
Polished treated	0	0.072 ± 0.001
	1	0.067 ± 0.005
	3	0.056 ± 0.004
	5	0.055 ± 0.004
	7	0.052 ± 0.004
	9	0.049 ± 0.004
Polished with plasma treated	0	0.072 ± 0.001
	1	0.066 ± 0.004
	3	0.052 ± 0.004
	5	0.047 ± 0.004
	7	0.044 ± 0.004
	9	0.041 ± 0.004

**Table 8:** Sizes of the inhibition zones of bacterial growth by orthodontic adhesives containing photoactivated 7.5 wt. % ZnO NPs/cCur, mm.

Microorganisms	Days							
	1	15	30	60	90	120	150	180
*S. mutans*	13	13	13	11	7	7	6	6
*S. sobrinus*	13	12	12	11	7	7	6	6
*L. acidophilus*	10	10	10	5	5	5	5	5

## Data Availability

Not applicable.

## Supplementary Materials

The following supporting information can be downloaded at: https://www.mdpi.com/article/10.3390/polym14050864/s1, Table S1: Antimicrobial action of nanoparticles and their fields of application in dentistry; Table S2: Major dental pathogens; Table S3: Minimum inhibitory concentration (MIC); Table S4: Surface roughness values (R_a_).

## Abbreviations

Ag NPs	silver nanoparticles
BGN	bioactive glass nanoparticles
BHI	brain heart infusion broth
CFU	colony-forming unit
Cs NPs	chitosan nanoparticles
CuO NPs	copper oxide nanoparticles
DNA	deoxyribonucleic acid
GIC	glass ionomer cement
HNTs	halloysite nanotubes
MDPC-23	odontoblast-like cell line
MIC	minimal inhibitory concentration
MgO NPs	magnesium oxide nanoparticles
nCaF_2_	calcium fluoride nanoparticles
L929	mouse fibroblast cell line
NCP	calcium phosphate nanoparticles
NDs	nanodiamonds
nHAps	hydroxyapatite nanoparticles
PMMA	poly(methyl methacrylate)
TiO_2_ NPs	titanium(IV) oxide nanoparticles
QND	quaternized nanodiamond
YFN	yttrium fluoride nanoparticles
ZnO NPs	zinc oxide nanoparticles
ZrO_2_ NPs	zirconium(IV) oxide nanoparticles
