# Supplementary material for: Polymeric Dental Nanomaterials: Antimicrobial Action
Source: Polymers (Basel). 2022 Feb 22;14(5):864. doi: 10.3390/polym14050864 (PMC8912874; doi:10.3390/polym14050864)
Supplement: Supplementary file 1 [file polymers-14-00864-s001.zip › polymers-1598367-supplementary-done.pdf]

polymers

MDPI

Supplementary Materials

Table S1. Antimicrobial action of nanoparticles and their fields of application in dentistry.

Nanoparticles	Antimicrobial action	Intended area of application	References
Silver nanoparticles	Activity against *Streptococcus mutans, Staphylococcus aureus* and *Escherichia coli* bacteria	Coatings for orthodontic appliances	[37]
	Activity against *Enterococcus faecalis* bacteria	Additives to endodontic sealer	[50]
	Activity against *Streptococcus mutans* bacteria	Components of toothpastes for the prevention of caries	[59]
	Inhibition of the growth of *Streptococcus mutans*, *Streptococcus salivarius* and *Lactobacillus acidophilus* bacteria	Additives to filling and restorative materials, adhesives for tooth restoration in dental therapy	[66]
	Inhibition of the growth of *Streptococcus mutans* bacteria	Fillers for dental restoration in dental therapy	[67]
	Activity against *Streptococcus mutans* bacteria	Components of self-etch adhesive system in dental therapy	[68]
	Activity against *Treponema denticola* bacteria	Additives to light-curing composite for orthodontic retainers	[69]
	Activity against *Staphylococcus aureus* and *Staphylococcus epidermidis* bacteria	Treatment of periodontal tissue diseases	[71]
	Activity against *Streptococcus mutans, Streptococcus sobrinus* and *Lactobacillus acidophilus* bacteria	Additives to orthodontic adhesives	[84], [85]
	Inhibition of the growth of biofilms cariogenic *Streptococcus mutans*, *Streptococcus sanguis*, *Lactobacillus acidophilus* bacteria	Components of composite resins used in fixed orthodontic retainers	[86]
	Activity against *Candida albicans* fungi	Additives to materials for the manufacture of dentures and dental implants	[91]
	Inhibition of the growth of biofilm *Candida glabrata* fungi	Additives to materials for the manufacture of dentures and dental implants	[92]
Nanodiamond	Activity against *Candida albicans* fungi	Component of resins for the manufacture of dentures	[51]
	Activity against anaerobic *Streptococcus mutans, Streptococcus mitis* and *Lactobacillus spp.* bacteria	Fillers of polymer fillings for one-stage caries treatment	[72]
	Inhibition of the growth of biofilm *Candida albicans* fungi	Component of orthodontic resins for the manufacture and repair of orthodontic appliances	[88]
	Inhibition of the growth of *Streptococcus mutans* bacteria	Component of resins for the manufacture of orthodontic attachments, brackets	[88]
	Reduction viability *Streptococcus mutans* bacteria	Component of resins for the manufacture of dentures	[94]

Zinc(II) oxide nanoparticles	Activity against *Streptococcus mutans* bacteria	Additives to restorative glass ionomer cements used in dental therapy	[41]
	Activity against *Streptococcus mutans, Enterococcus faecalis* and *Lactobacillus fermentum* bacteria, and *Candida albicans* fungi	Treatment of caries in dental therapy and endodontics	[73]
	Activity against *Porphyromonas gingivalis* and *Actinomyces naeslundii* bacteria	Additives to sealants for endodontic treatment of apical periodontitis	[74]
	Activity against *Streptococcus mutans* and *Lactobacillus acidophilus* bacteria	Components of adhesives for tooth restoration in dental therapy	[75]
	Inhibition of the metabolic activity of biofilms cariogenic *Streptococcus mutans, Streptococcus sobrinus* and *Lactobacillus acidophilus* bacteria after photoactivation	Components of orthodontic adhesive for fixing orthodontic appliances	[90]
Titanium(IV) oxide nanoparticles	Activity against *Streptococcus mutans* bacteria	Components of orthodontic adhesive for fixing orthodontic appliances	[26]
	Activity against *Streptococcus mutans* bacteria	Additives to light-curing adhesives for dental restorations	[52]
	Activity against *Staphylococcus aureus* and *Escherichia coli* bacteria	Fillers for maxillofacial prostheses	[98]
Zirconium(IV) oxide nanoparticles	Activity against *Streptococcus mutans* and *Streptococcus sobrinus* bacteria	Fillers for filling and restorative materials in dental therapy	[77], [78]
Hydroxyapatite nanoparticles	Enamel remineralization in caries-like enamel lesions induced by *Streptococcus mutans* biofilm	Fillers for filling and restorative materials in dental therapy	[79]
	Activity against *Enterococcus faecalis* and *Streptococcus mitis* bacteria	Components of root canal filling materials	[80]
Bioactive glass nanoparticles	Activity against gram-positive bacteria *Streptococcus mutans*	Components of orthodontic adhesive for fixing orthodontic appliances	[58]
Copper (II) oxide nanoparticles	Inhibition of the growth of *Candida albicans* fungi	Components of soft-denture liners	[61]
	Inhibition of the growth of *Candida dubliniensis* fungi, *Streptococcus salivarius, Streptococcus sanguis, Streptococcus mutans* bacteria	Components of partial and complete removable dentures	[98]
Yttrium(III) fluoride nanoparticles	Inhibition of the growth of *Streptococcus mutans*	Components of orthodontic composite resins	[58]
Calcium fluoride nanoparticles	Remineralization ability to protect enamel under biofilm *Streptococcus mutans* bacteria	Fillers for filling and restorative materials in dental therapy	[59]
Calcium phosphate nanoparticles	Remineralization ability to protect enamel under biofilm *Streptococcus mutans* bacteria	Fillers for filling and restorative materials in dental therapy	[83]
Chitosan nanoparticles	Inhibition of the growth of *Candida albicans* fungi, *Streptococcus mutans, Pseudomonas aeruginosa* and *Enterococcus faecalis* bacteria	Components of tissue conditioner	[93]
Halloysite nanotubes	Inhibition of the growth of *Candida albicans* fungi	Components of partial and complete removable dentures	[97]

Table S2. Major dental pathogens.

Microorganism	Pathology caused by a microorganism
S. mutans	Caries, apical periodontitis, peri-implantitis
E. faecalis	Apical periodontitis
S. mitis	Caries, peri-implantitis
Lactobacillus spp.	Caries
P. gingivalis	Apical periodontitis, periodontitis, peri-implantitis
A. naeslundii	Apical periodontitis, periodontitis
S. aureus	Caries, periodontitis, peri-implantitis
S. salivarius	Caries, peri-implantitis
L. acidophilus	Caries
C. albicans	Oral candidosis, prosthetic stomatitis
C. glabrata	Oral candidosis, prosthetic stomatitis
C. dubliniensis	Oral candidosis, prosthetic stomatitis
S. sanguis	Caries, periodontitis
T. denticola	Periodontitis
S. sobrinus	Caries
E. coli	Caries

Table S3. Minimum inhibitory concentration (MIC).

Nanoparticles	Microorganisms	MIC, μg mL^{-1}	References
CuO NPs (18.3 nm)	*C. albicans*	64	[61]
	S. mutans	128	
	S. sobrinus	128	
	S. salivarius	64	
Ag NPs	*S. mutans*	12.5	[68]
ZnO NPs (20 nm)	*S. mutans*	0.625	[73]
	E. faecalis	0.312	
	L. fermentum	5	
	C. albicans	10	
ZnO NPs (40 nm)	*S. mutans*	1.04	[73]
	E. faecalis	0.625	
	L. fermentum	10	
	C. albicans	>10	
ZnO NPs (140 nm)	*S. mutans*	1.66	[73]
	E. faecalis	1.25	
	L. fermentum	>10	
	C. albicans	>10	
ZnO NPs (10 nm)	*P. gingivalis*	10	[74]
	A. naeslundii	40	
MgO NPs (20 nm)	*S. mutans, S. sobrinus*	500	[78]
ZnO NPs (38.19 nm)	*S. mutans*	32	[81]
Cs NPs (82.87 nm)	*S. mutans*	2048	
ZnO NPs + Cs NPs (185.7 nm)	*S. mutans*	1024	
TiO_2 NPs (17 nm)	*S. salivarius, S. sanguis, C. dubliniensis*	32-64	[97]
CuO NPs (40 nm)			

Table S4. Surface roughness values (R_a).

Materials	Surface roughness, nm	References
Heat-polymerized (control)	150±10	[44]
Acrylic resin reinforced with 0.5 wt.% ND	60±10	
Heat-cured PMMA (control)	150±10	[45]
Heat-cured PMMA + 5 wt. % ZrO_2 NPs	200±10	
Heat-polymerized acrylic resin Major base 20 (control)	129±11	[51]
Heat-polymerized acrylic resin Major base 20 + 0.5 wt. % ND	39±9	
Composite resin Filtek Z350 XT (control)	190±10	[76]
Composite resin Filtek Z350 XT + 2 wt. % TiO_2 NPs	220±10	
Orthodontic acrylic resin system Ortho-Jet (control)	146.792±22.06	[88]
Orthodontic acrylic resin system Ortho-Jet + 0.1 wt. % ND	162.502±4.24	
Acrylate-based resin PMMA material (control)	470±110	[89]
Acrylate-based resin PMMA material + 0.1 wt. % ND powder	480±160	
Untreated PMMA (control)	123±15	[90]
PMMA + 3 wt. % Ag NPs	61±6	
PMMA + 3.5 wt. % Ag NPs	41±3	
Untreated PMMA (control)	142±38	[96]
PMMA + 1 wt. % TiO_2 NPs	65±15	
PMMA + 3 wt. % TiO_2 NPs	40±10	
PMMA + 1 wt. % HNTs	38±10	
PMMA + 3 wt. % HNTs	15±2	
Neat silicone (control)	567±10.8	[100]
Silicone + 1 % wt. ZrO_2 nanopowder	570±6.3	
